# Fabrication of Europium-Doped CaF_2_ Films via Sol-Gel Synthesis as Down-Shifting Layers for Solar Cell Applications

**DOI:** 10.3390/ma16216889

**Published:** 2023-10-27

**Authors:** Anna Lucia Pellegrino, Emil Milan, Adolfo Speghini, Graziella Malandrino

**Affiliations:** 1 Dipartimento di Scienze Chimiche, Università di Catania and INSTM UdR Catania, Viale A. Doria 6, I-95125 Catania, Italy; annalucia.pellegrino@unict.it; 2 Nanomaterials Research Group, Dipartimento di Biotecnologie, Università di Verona and INSTM UdR Verona, Strada le Grazie 15, I-37134 Verona, Italy; emil.milan@univr.it (E.M.); adolfo.speghini@univr.it (A.S.)

**Keywords:** metalorganic complexes, morphological control, luminescence, energy conversion

## Abstract

In the present work, an in-depth study on the sol-gel process for the fabrication of Eu-doped CaF_2_ materials in the form of thin films has been addressed for the production of down-shifting layers. Fine-tuning of the operative parameters, such as the annealing temperature, substrate nature and doping ion percentage, has been finalized in order to obtain Eu(III)-doped CaF_2_ thin films via a reproducible and selective solution process for down-shifting applications. An accurate balance of such parameters allows for obtaining films with high uniformity in terms of both their structural and compositional features. The starting point of the synthesis is the use of a mixture of Ca(hfa)_2_•diglyme•H_2_O and Eu(hfa)_3_•diglyme adducts, with a suited ratio to produce 5%, 10% and 15% Eu-doped CaF_2_ films, in a water/ethanol solution. A full investigation of the structural, morphological and compositional features of the films, inspected using X-ray diffraction analysis (XRD), field emission scanning electron microscopy (FE-SEM) and energy dispersive X-ray analysis (EDX), respectively, has stated a correlation between the annealing temperature and the structural characteristics and morphology of the CaF_2_ thin films. Interestingly, crystalline CaF_2_ films are obtained at quite low temperatures of 350–400 °C. The down-shifting properties, validated by taking luminescence measurements under UV excitation, have allowed us to correlate the local environment in terms of the degree of symmetry around the europium ions with the relative doping ion percentages.

## 1. Introduction

Recently, great effort has been devoted to the development of innovative materials with high impact in an energetic scenario. In particular, the possibility of engineering new devices with increasing efficiency of energy production represents the main challenge in the photovoltaic field [1,2,3]. Among a plethora of inorganic functional materials, fluoride-based systems are one of the most promising classes in this field due to their excellent chemical stability and optical properties [4,5], which allow them to extend their uses in a wide range of microelectronic, photonic and nanomedicine applications [6,7,8]. Particularly, the doping of fluoride inorganic systems with luminescent species, such as trivalent lanthanide ions (Ln^3+^), represents one of the most intriguing strategies to obtain more efficient photovoltaic devices through energy conversion processes [9,10]. This class of materials combines the excellent optical properties of the fluoride matrices, such as low phonon energy and high optical transparency [11,12], with the energy conversion properties of the doping ions both as down- and up-conversion systems. In fact, appropriate combinations of luminescent lanthanide ions, such as Yb^3+^/Er^3+^ or Yb^3+^/Tm^3+^ for up-conversion (UC) and Eu^3+^ for down-conversion/down-shifting (DC and DS) systems, are able to collect the radiation outside of the absorption range of the active photovoltaic material and shift its energy to a more suitable optical region, with the aim of enhancing the efficiency of solar devices. Specifically, in UC processes, photons in the near-infrared region are converted into higher-energy photons, i.e., in the ultraviolet (UV) or in the visible regions; in DC processes, one high-energy photon is converted into two or more photons at lower energy and in the DS phenomena, high-energy photons, usually in the UV region, are absorbed and converted into lower-energy photons [13]. Among the several DS species, europium (III) is one of the most promising down-shifters, due to its high emission efficiency and long lifetimes [14,15,16], thus allowing the exploitation of the UV range of the solar spectrum. Furthermore, the inorganic host component also plays a crucial role in the engineering of efficient energy conversion systems. The most extensively investigated inorganic matrices are represented by binary and multicomponent fluorides of the type CaF_2_, BaF_2_ and SrF_2_ or NaYF_4_, NaGdF_4_ and LiYF_4_ [17,18,19,20,21,22]. Among them, calcium fluoride has been regarded as one of the most efficient matrices for lanthanide ions in energy conversion systems, due to its optical properties [23,24] and chemical stability. However, calcium fluoride has been reported in the literature in the form of lanthanide-doped nanoparticles for applications in nanomedicine [25], while in the form of thin films, it has been much less investigated. Nevertheless, the bottleneck for its massive application in optics, microelectronics and the PV field is related to the possibility of fabrication process development, which allows for the growth of nanostructured doped fluoride systems in the form of thin film with a wide range of structural, morphological and compositional control. Up to now, the most explored synthetic routes for pure and doped CaF_2_ are related to vapor deposition growth, such as electron beam evaporation, molecular beam epitaxy, chemical vapor deposition and solution routes [26,27,28,29].

In this scenario, the present sol-gel approach represents the first report on a cheap and industrially appealing solution process for the synthesis of compact and homogenous Eu-doped CaF_2_ thin film with high structural and compositional tunable properties. In particular, we report an in-depth study on a combined sol-gel/spin-coating approach to the fabrication of a down-shifting layer based on an Eu-doped CaF_2_ thin film starting from a mixture of Ca(hfa)_2_•diglyme•H_2_O and Eu(hfa)_3_•diglyme complex in a water/ethanol solution. The annealing temperature, substrate nature and doping europium percentage are the key parameters to fine-tune both the morphological and luminescence features of the as-synthesized CaF_2_-based films. A complete investigation of the structural, morphological and compositional characterization of the Eu-doped CaF_2_ thin film has been executed using X-ray diffraction analysis (XRD), field emission scanning electron microscopy (FE-SEM) and energy dispersive X-ray analysis (EDX), respectively. Finally, the luminescent properties as down-shifting layers as a function of europium ion concentration have been investigated using luminescence measurements.

## 2. Materials and Methods

### 2.1. CaF_2_: Eu Synthesis

The Ca(hfa)_2_•diglyme•H_2_O and Eu(hfa)_3_•diglyme precursors were synthesized as previously reported in [14,30].

The sol–gel reaction was conducted in a water/ethanol solution with trifluoroacetic acid as a catalyst for the hydrolysis reactions, starting from a mixture of Ca and Eu precursors. The adducts were mixed in molar ratio values of 0.95:0.05, 0.9:0.1 and 0.85:0.15 mmol for the preparation of 5%, 10% and 15% of Eu-doping CaF_2_ films, respectively. The reaction and the different molar ratios used are reported in the following:

0.95 Ca(hfa)_2_•diglyme•H_2_O:0.05 Eu(hfa)_3_•diglyme:87 C_2_H_5_OH:3 H_2_O:0.8 CF_3_COOH for CaF_2_:Eu (5%).
0.90 Ca(hfa)_2_•diglyme•H_2_O:0.10 Eu(hfa)_3_•diglyme:87 C_2_H_5_OH:3 H_2_O:0.8 CF_3_COOH for CaF_2_:Eu (10%).
0.85 Ca(hfa)_2_•diglyme•H_2_O:0.15 Eu(hfa)_3_•diglyme:87 C_2_H_5_OH:3 H_2_O:0.8 CF_3_COOH for CaF_2_:Eu (15%).

The different solutions were submitted to hydrolysis and aging reactions under stirring at 60 °C for 20 h. The spin-coating process was carried out on Si (100) and glass substrates about 1 cm × 2 cm, cut from microscope glass slides (Forlab, Carlo Erba, Milan, Italy) of 0.8 mm thickness. The films were deposited via spin-coating using a multistep approach with a four-time deposition. After each spin, a 10 min annealing step at 350 °C or 400 °C in air was carried out. Finally, the films were annealed at 350 °C or 400 °C in air for 1 h. The spin-coating was carried out with a ramping rate of 1000 revolutions per minute (rpm), a spinning rate of 3000 rpm and a time of 60 s using a Spincoater SPIN150 (SPS Europe, Putten, The Netherlands) system.

### 2.2. Characterization

The film structures were analyzed using XRD in grazing incidence mode (0.5°) and using a SmartLab Rigaku diffractometer (Rigaku, Tokyo, Japan) operating at 45 kV and 200 mA, equipped with a rotating anode of Cu K_α_ radiation. The film morphologies were analyzed via FE-SEM using a ZEISS SUPRA 55 VP field emission microscope (ZEISS, Jena, Germany). For the FE-SEM characterization, the films deposited onto Si were analyzed as prepared, while a very thin Au layer was sputtered onto the films and deposited onto a non-conducting substrate such as glass. Energy-dispersive X-ray (EDX) analysis allowed the atomic compositional analysis of the samples. An Oxford INCA windowless detector (Oxford Instruments, Abingdon, UK), having a resolution of 127 eV as the full-width half maximum (FWHM) of the Mn K_α_, was used. For measuring the luminescence spectra, the samples were excited using an LED flashlight and a band-pass filter centered at 390 nm. The emission spectra were measured using a 4× microscopy objective at a 90° geometry and an edge filter at 532 nm (Semrock 532 nm RazorEdge^®^ ultrasteep, IDEX Health & Science, LLC, Rochester, NY, USA) to reject the exciting radiation. The emission spectra were dispersed using a single monochromator (Andor Shamrock 500i, 300 lines/mm grating, Andor technology, Belfast, Northern Ireland) with an optical resolution of 1.3 nm and measured with a Peltier-cooled (−90 °C) CCD camera (Andor, iDus, Andor technology, Belfast, Northern Ireland).

## 3. Results and Discussion

An optimized procedure of sol-gel reaction and spin-coating deposition was applied for the fabrication of Eu-doped CaF_2_ thin films. Particularly, the operative parameters of the process, such as the annealing temperature, nature of the substrate and europium concentration were finely tuned in order to obtain full control of the structural, morphological and luminescent properties of the down-shifting layers. It is worth noting that the present work represents, to the best of our knowledge, the first report on the fabrication of down-shifting CaF_2_ thin films using a sol-gel process. Similar synthetic strategies have been reported in the last years for the fabrication of pure binary fluoride CaF_2_ and the up-converting systems of CaF_2_: Yb^3+^, Er^3+^, Tm^3+^ [28] and multicomponent fluoride NaYF_4_: Yb^3+^, Er^3+^ and NaYF_4_: Yb^3+^, Tm^3+^ [20]. The approach herein represents an easy synthetic method for producing Eu-doped CaF_2_ films with inexpensive equipment and high crystallinity. Furthermore, the use of Ca(hfa)_2_•diglyme•H_2_O and Eu(hfa)_3_•diglyme adducts, as a starting mixture, provides a unique source for the Ca, Eu and F components.

### 3.1. Structural Investigation

**Figure 1:**
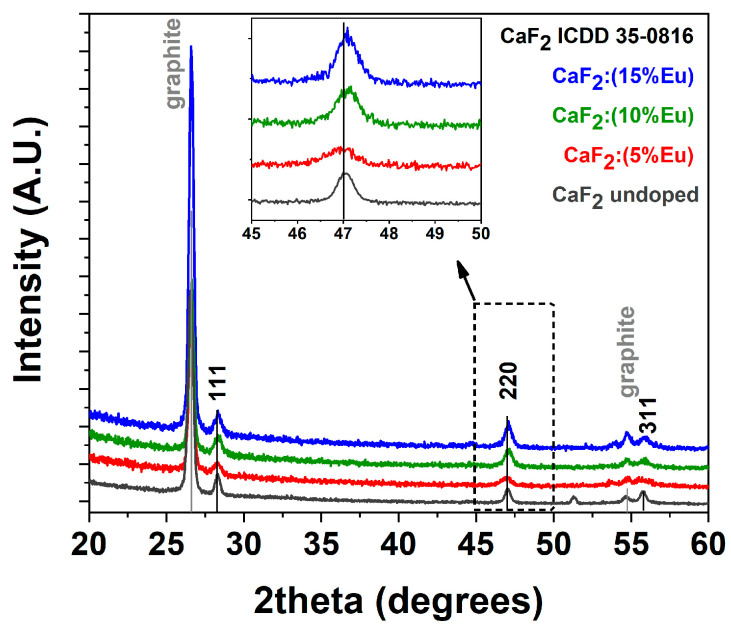
XRD patterns of undoped CaF_2_ (gray line), CaF_2_: Eu (5%) (red line), CaF_2_: Eu (10%) (green line) and CaF_2_: Eu (15%) (blue line) thin films on Si (100) substrate at the annealing temperature of 400 °C. Graphite is used as an internal standard.

Structural characterization of the prepared films at different doping ion percentages was conducted using X-ray diffraction (XRD) analysis and has been reported in Figure 1. The patterns show the formation of pure and nanostructured CaF_2_ as confirmed by the presence of characteristic peaks at 2θ = 28.29, 47.09 and 55.81° corresponding to reflections of the (111), (220) and (311) lattice planes, respectively, in accordance with the ICDD card n.35-0816 of the CaF_2_ phase. As a reference, also an undoped CaF_2_ sample obtained at 400 °C on a silicon substrate has been reported, confirming a perfect match with the ICDD card of the pure CaF_2_ phase. The small peak around 51° arises from the machine when measurements are carried out in grazing incidence. Furthermore, an in-depth investigation was conducted with the use of graphite as an internal standard for each sample in order to evaluate the peak position and the potential shift of the CaF_2_ signals. A comparison of the three patterns reported as a function of the Eu-doping concentrations displays some differences in terms of the peak positions with respect to those reported in the diffraction card (black lines in Figure 1). In particular, a magnification of the 220 reflection, shown in the inset of Figure 1, displays a perfect match of the peak arising from the CaF_2_: Eu (5%) sample in comparison with the position reported in the ICDD database for the CaF_2_ phase, and a slight shift toward higher angles for CaF_2_: Eu (10%) and CaF_2_: Eu (15%).

This behavior can be rationalized considering the smaller ionic radius of the Eu-doping ion, which is assumed to be in a substitutional position with respect to Ca^2+^. In fact, Eu^3+^ in an eight coordination has an ionic radius of 1.066 Å, which is slightly smaller than the Ca^2+^ ionic radius of 1.12 Å for the same coordination [31]. This effect results in a slight contraction of the lattice structure at a higher doping ion concentration and thus in a shift toward the higher angles of the diffraction peaks. Notably, considering the need for charge balance due to the different charges, 2+ for calcium and 3+ for europium, both the formation of interstitial fluoride ions or clusters can be possible [17,32,33].

### 3.2. Morphological Characterization

**Figure 2:**
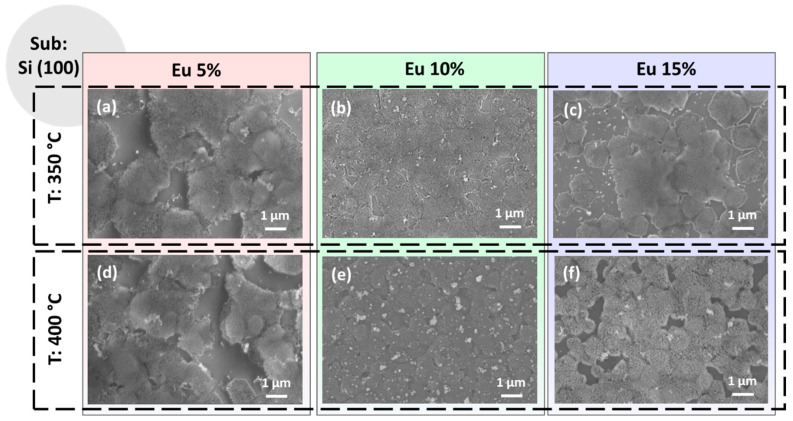
FE-SEM images of the CaF_2_ films deposited onto Si (100) substrate at (**a**) 350 °C and 5% Eu doping; (**b**) 350 °C and 10% Eu doping; (**c**) 350 °C and 15% Eu doping; (**d**) 400 °C and 5% Eu doping; (**e**) 400 °C and 10% Eu doping and (**f**) 400 °C and 15% Eu doping.

**Figure 3:**
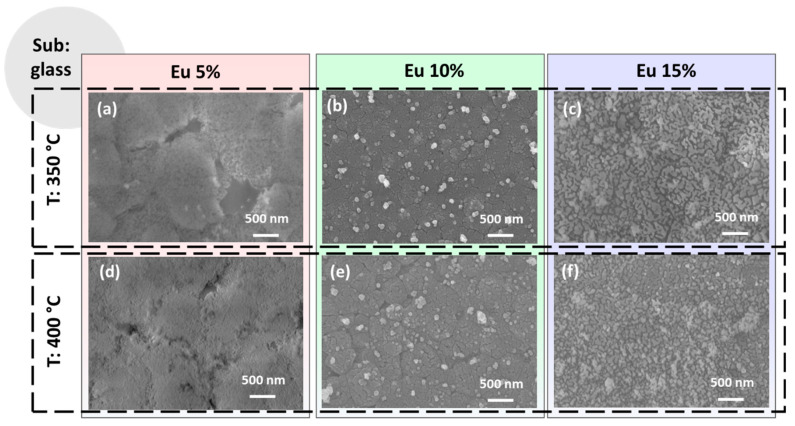
FE-SEM images of the CaF_2_ films deposited onto glass substrate at (**a**) 350 °C and 5% Eu doping; (**b**) 350 °C and 10% Eu doping; (**c**) 350 °C and 15% Eu doping; (**d**) 400 °C and 5% Eu doping; (**e**) 400 °C and 10% Eu doping and (**f**) 400 °C and 15% Eu doping.

The different conditions of the synthetic process affect the morphology of the films both in terms of the uniformity of the substrate coverage and in the general aspect of the layers. In particular, a full overview of the different morphologies obtained for the Eu-doped CaF_2_ films has been reported for the Si substrates in Figure 2 and for the glass substrates in Figure 3.

The FE-SEM images of the CaF_2_: Eu (5%) films on the Si (100) substrate are reported in Figure 2a,d for the two annealing temperatures 350 °C and 400 °C, respectively. The images display, for both samples, similar morphologies with the formation of discontinuous coverage being independent from the annealing temperature. The growth is quite porous and the presence of regions not covered could be attributed to two aspects: (i) the density of the incipient gel used in the spin-coating deposition for this Ca: Eu ratio and (ii) the poor wettability of the silicon substrate. Therefore, under these operative parameters, the annealing temperature slightly affects the morphology of the films. The observed morphology may be compared to the sponge-like or coral-like shape observed for the CaF_2_ nanostructures prepared using pulsed electron beam evaporation and thermally annealed at 200 °C [26]. On the other hand, the morphology is completely different from that observed in the MBE-grown CaF_2_ films, where nanocrystals are derived from epitaxial growth [26], or from the liquid phase epitaxy grown layers, where the CaF_2_ films were deposited onto a CaF_2_ single-crystal substrate [29].

In Figure 2b,e are shown the CaF_2_: Eu (10%) films on Si (100) at 350 °C and 400 °C annealing temperatures, respectively. Under these process parameters, the films appear more compact and homogenous, even if some outgrowths are visible and are more evident in Figure 2e for the sample obtained at 400 °C. This feature has been already observed for pure CaF_2_ film growth on Si [28] and could arise during the annealing step process. Finally, the samples with the highest percentage of Eu-doping ions, i.e., CaF_2_: Eu (15%), are reported in Figure 2c,f for the two annealing temperatures. They display partial coverage of the silicon substrates and the presence of some outgrowths similar to those found in Figure 2b,e, but aggregated with each other. However, in these last two samples, the coverage of the Si substrate, even if it appears somewhat similar to what we have observed for the CaF_2_: Eu (5%) films (Figure 2a,d), presents a more compact and flat surface, especially for the sample in Figure 2f. This last aspect could be associated with the higher treatment temperature, i.e., 400 °C, used in these last films.

The morphologies obtained on glass substrates are presented in the overview in Figure 3 as a function of the annealing temperatures and Eu-doping percentage. The CaF_2_: Eu (5%) films on glass obtained at 350 °C and 400 °C shown in Figure 3a,d present both a very flat and smooth morphology, similar to the one observed for the same film composition on the Si substrate (see Figure 2a,d). However, for these samples, the coverage seems to be more compact, with only a few voids for the sample at 350 °C in Figure 2a. This tendency is likely due to the better wettability of the glass substrate compared to silicon, which seems the main aspect in determining the morphology features of the samples in this case. The CaF_2_: Eu (10%) films on glass annealed at 350 °C and 400 °C, respectively, are reported in Figure 2b,e. For both these systems, we observe a similar morphology, regardless of the annealing temperature, with a uniform and compact layer characterized by the presence of some outgrowths being uniformly distributed on the surfaces. These features are very similar to what we found for the analogous films obtained on silicon (see Figure 2b,e). Finally, the CaF_2_: Eu (15%) films on glass are displayed in Figure 3c,f for 350 °C and 400 °C annealing treatments. For these samples, we observe a different morphology characterized by homogeneous coating with small grains in the order of tens of nanometers barely visible. This morphology suggests grain coalescence phenomena in the coating formation during the annealing treatment, as already observed in similar conditions for the up-converting CaF_2_: Yb^3+^, Er^3+^ and Tm^3+^ layers [28]. Notably, at higher temperatures (see Figure 3f), the films appear more compact and uniform. Therefore, the different morphologies observed using FE-SEM analyses can be ascribed to many factors: (i) the different wettability of the substrates, which is the main reason for the films obtained on silicon; (ii) the density of the sol used, which is directly correlated with the starting mixture of the Ca and Eu adducts in the gel; (iii) a slight variation in the spin-coating step; (iv) annealing temperature of the final treatments, as confirmed by the trend of the best uniformity being observed at a higher temperature. In each case, the balance of all these aspects results in a plethora of different morphologies.

**Figure 4:**
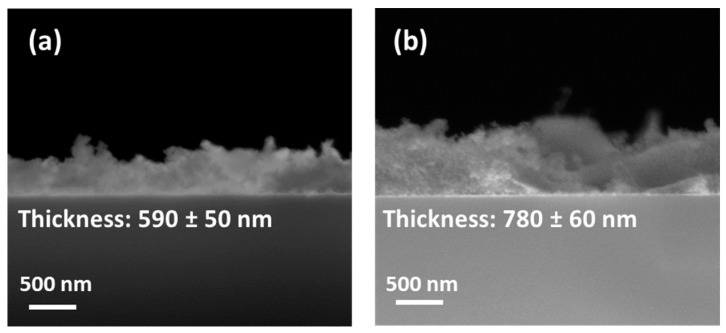
FE-SEM images in cross-section of CaF_2_: Eu (10%) films deposited onto Si (100) at (**a**) 350 °C and (**b**) 400 °C.

Then, the thickness of the as-prepared films was evaluated using FE-SEM analysis in cross-sectional mode. The images of the CaF_2_: Eu (10%) samples on silicon at 350 °C and 400 °C, for which plain-view analyses reveal more uniform and compact films, have been reported in Figure 4.

At a lower annealing temperature, i.e., 350 °C, the cross-sectional image in Figure 4a confirms the formation of a compact layer with a thickness of about 590 ± 50 nm. The CaF_2_: Eu (10%) sample obtained at the higher temperature of 400 °C in Figure 4b displays a similar compact and uniform film of 780 ± 60 nm.

The increment in the thickness value at a higher annealing temperature can be explained considering that already during the deposition step, which occurs via a multistep procedure consisting of four-time spin-coating depositions alternated with fast annealing in the air for 10 min, the crystallization of the layers takes place. The deposition process hence is more efficient at higher annealing temperatures, allowing the formation of slightly thicker films.

### 3.3. Compositional Characterization

**Figure 5:**
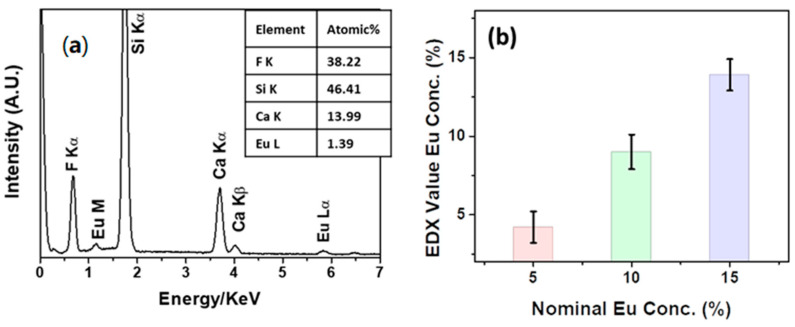
(**a**) EDX spectrum of the CaF_2_: Eu (10%) film deposited onto Si (100) at 400 °C and (**b**) relationship between nominal Eu concentrations in the Eu-doped CaF_2_ films deposited onto Si (100) at 400 °C and the values extrapolated from EDX quantitative analyses.

In order to ensure the down-shifting properties of the Eu-doped CaF_2_ films, a preliminary study on the sample composition has been conducted using energy dispersive X-ray (EDX) analysis. In Figure 5a, the EDX spectrum of the CaF_2_: Eu (10%) film deposited onto Si (100) at 400 °C is reported as a case study. The spectrum shows the K_α_ peak of fluorine, the K_α_ peak of the silicon substrate and the K_α_ and K_β_ peaks of calcium, at values of 0.67, 1.74, 3.70 and 4.02 keV, respectively. In addition, the presence of europium is confirmed by the signals at 1.14 and 5.81 keV, related to the M and L lines, respectively. Notably, the absence of C and O elements in the spectrum points to the good reactivity and clean decomposition of the Ca and Eu precursors during the sol-gel process. The quantitative analysis shown in the inset in Figure 5a confirms the correct stoichiometry of the Eu concentration of the film at about 10%, which is coincident with the value set in the starting mixture, i.e., 0.90: 0.10 of Ca(hfa)_2_•diglyme•H_2_O: Eu(hfa)_3_•diglyme.

Additionally, quantitative analysis was conducted for all the Eu-doped CaF_2_ samples deposited onto silicon and is reported in the graph in Figure 5b. Several different regions for each kind of samples have been analyzed in order to ensure both a more representative value of Eu concentration and a good homogeneity of the layers. The graph displays the average value of the Eu concentration recorded using EDX quantitative analysis versus the nominal concentration used in the starting mixture of each Eu-doped CaF_2_ sample. Concentrations of 4.2 ± 1%, 9.0 ± 1.1% and 13.9 ± 1.0% have been obtained for the CaF_2_: Eu (5%), CaF_2_: Eu (10%) and CaF_2_: Eu (15%) films, respectively, confirming a good match between the nominal and experimental Eu-doping concentrations.

**Figure 6:**
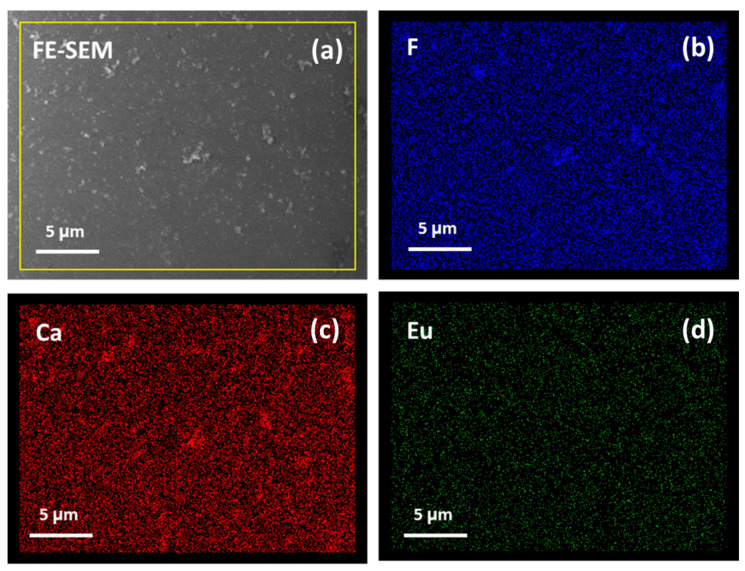
FE-SEM images (**a**) and EDX map analysis of F (**b**), Ca (**c**) and Eu (**d**) elements for the CaF_2_: Eu (10%) film deposited onto Si (100) at 400 °C.

Finally, the homogeneity of the systems has been evaluated using EDX map analysis over a large area. In particular, for the CaF_2_: Eu (10%) onto Si at 400 °C sample, the maps of the F, Ca and Eu elements are reported in Figure 6. Notably, the doping ion distribution is very uniform over the analyzed area (about 20 × 28 μm), confirming the suitability of the synthetic process for the fabrication of down-converting system with good luminescence performance.

### 3.4. Luminescence Properties

**Figure 7:**
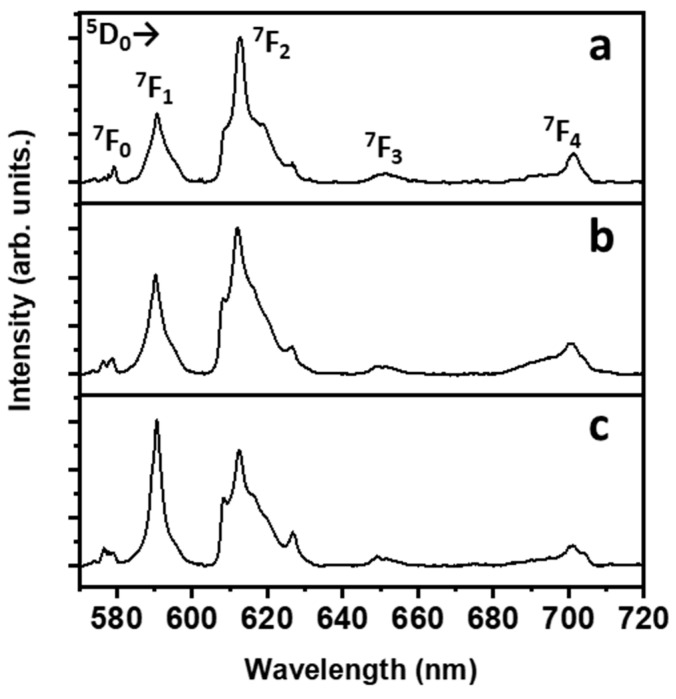
Room temperature emission spectra with transition assignments of the CaF_2_ thin films deposited onto glass substrate at 400 °C at different Eu^3+^ ion concentrations: (**a**) 5%; (**b**) 10%; (**c**) 15%. All transitions originate from the ^5^D_0_ level. The end level is shown in the picture close to the corresponding emission band.

Radiation centred around 390 nm was chosen as the excitation source because at this wavelength, there is a strong absorption by Eu^3+^ ions due to the ^7^F_0_→^5^L_6_ transition. It is worth noting that the oscillator strength of this transition is among the strongest ones for Eu^3+^ ions in the CaF_2_ host in the UV-visible range [32]. The room temperature emission spectra of the Eu^3+^-doped CaF_2_ films deposited onto the glass substrate at 400 °C are shown in Figure 7. The observed emission bands are due to transitions of the Eu^3+^ ions, ranging from the ^5^D_0_ excited level to the ^7^F_J_ multiplet (J = 0, 1, 2, 3, 4). In particular, the bands assigned to the ^5^D_0_→^7^F_1_ (in the 580–600 nm range) and ^5^D_0_→^7^F_2_ transitions (in the 605–630 nm) dominate the emission spectrum for all the samples. The emission spectra are compatible with those observed for similar samples, e.g., CaF_2_: Eu^3+^ nanoparticles prepared using a hydrothermal technique [34] and using a sol-gel technique [35], Eu^3+^-doped CaF_2_ thin films prepared using electrochemical processing [36] and thin films of Eu^3+^-doped CaF_2_ deposited onto an aluminum layer using a vacuum deposition approach [37]. It has to be noted that the ^5^D_0_→^7^F_1_ transition is a magnetic dipole one, not dependent on the local environment around the lanthanide ion [38]. On the other hand, the ^5^D_0_→^7^F_2_ transition is an electric dipole, allowed only for lanthanide sites without inversion symmetry. This property makes the latter transition highly sensitive to probing the local environment of the Eu^3+^ ions. The ratio between the intensities of the bands due to the two abovementioned transitions is useful to get insights about the degree of asymmetry in the local environment of the lanthanide ion [39]. The asymmetry ratio *R* is defined as:

(1) R=ID05→F27ID05→F17

where *I* is the integrated area of the ^5^*D*_0_→^7^*F_J_* (*J* = 1, 2) transitions. The *R* values increase on decreasing the degree of symmetry around the lanthanide ion. The calculated *R* values for the samples are 3.12, 2.70 and 1.92 for Eu^3+^ concentrations of 5%, 10% and 15%, respectively. A decrease in the *R* values is observed on increasing the lanthanide concentration, indicating that on average, the symmetry of the lanthanide ions accordingly increases. It is well known that lanthanide ions can be accommodated in the CaF_2_ structure in several sites with different symmetries [40,41,42]. In the present case, an increased occupation of more symmetric sites on increasing the lanthanide concentration is compatible with the observed spectra, inducing an overall increase in the symmetry around the lanthanide ions and therefore a decrease in the *R* value. For instance, on increasing the lanthanide concentration, a higher number of Eu^3+^ ions could substitute the regular Ca^2+^ lattice sites and fewer Eu^3+^ ions could occupy the particle surface or defect sites [43].

Any possibility of the presence of Eu^2+^ can be excluded since no peaks related to the Eu^2+^ species are observed in the spectrum. This outcome may be compared to the findings reported in the study by Secu et al. [44], who synthesized Eu(II)-doped CaF_2_ films using sol-gel and annealing techniques under a reduced atmosphere, while the present thermal treatments are carried out in air.

## 4. Conclusions

In conclusion, an in-depth study is herein described for the fabrication of europium-doped CaF_2_ systems in the form of thin films using a combined sol-gel/spin-coating deposition process. To the best of our knowledge, the present work is the first report on the production of Eu^3+^-doped CaF_2_-based thin films using a sol-gel approach. Specifically, through fine-tuning the operative parameters, such as the annealing temperature, substrate nature and doping ion concentration, we have managed to optimize the formation of homogeneous films of CaF_2_ on both silicon and glass substrates. XRD characterization assessed that crystalline films are obtained with a thermal treatment of 350–400 °C on both substrates. EDX microanalyses confirmed the film purity since neither C nor O is observed in the spectrum, pointing to a clean decomposition of the precursor, and the europium percentage in the film is parallel with the nominal amount in the initial sol mixture. Actually, the sol-gel process combined with a spin-coating deposition represents an appealing method at an industrial level due to the low-cost equipment involved and the relatively low temperature of the annealing treatment. In alternative to spin-coating, spraying could be applied for a potential scaling-up of the process. Finally, the luminescence properties of the samples have been investigated by taking spectroscopy measurements under UV excitation. The down-shifting features have confirmed the functional properties of the material and thus its potential use in solar cell devices.

## Data Availability

Data are available from the authors upon request.
